# Texture-based classification of different single liver lesion based on SPAIR T2W MRI images

**DOI:** 10.1186/s12880-017-0212-x

**Published:** 2017-07-13

**Authors:** Zhenjiang Li, Yu Mao, Wei Huang, Hongsheng Li, Jian Zhu, Wanhu Li, Baosheng Li

**Affiliations:** Shandong Cancer Hospital to Shandong University, Shandong Academy of Medical Sciences, Jinan, Shandong China

**Keywords:** Liver carcinoma, Texture analysis, Magnetic resonance imaging, SPAIR T2-weighted imaging

## Abstract

**Background:**

To assess the feasibility of texture analysis (TA) based on spectral attenuated inversion-recovery T2 weighted magnetic resonance imaging (SPAIR T2W-MRI) for the classification of hepatic hemangioma (HH), hepatic metastases (HM) and hepatocellular carcinoma (HCC).

**Methods:**

The SPAIR T2W-MRI data of 162 patients with HH (n=55), HM (n=67) and HCC (n=40) were retrospectively analyzed. We used two independent cohorts for training (n = 112 patients) and validation (n = 50 patients). The TA was performed and textual parameters derived from the gray level co-occurrence matrix (GLCM), gray level gradient co-occurrence matrix (GLGCM), gray-level run-length matrix (GLRLM), Gabor wavelet transform (GWTF), intensity-size-zone matrix (ISZM), and histogram features were calculated. The capacity of each parameter to classify three types of single liver lesions was assessed using the Kruskal-Wallis test. Specificity and sensitivity for each of the studied parameters were derived using ROC curves. Four supervised classification algorithms were trained with the most influential textural features in the classification of tumor types. The test datasets validated the reliability of the models.

**Results:**

The texture analyses showed that the HH versus HM, HM versus HCC, and HH versus HCC could be differentiated by 9, 16 and 10 feature parameters, respectively. The model’s misclassification rates were 11.7, 9.6 and 9.7% respectively. No texture feature was able to adequately distinguish among the three types of single liver lesions at the same time. The BP-ANN model had better predictive ability.

**Conclusion:**

Texture features of SPAIR T2W-MRI can classify the three types of single liver lesions (HH, HM and HCC) and may serve as an adjunct tool for accurate diagnosis of these diseases.

**Electronic supplementary material:**

The online version of this article (doi:10.1186/s12880-017-0212-x) contains supplementary material, which is available to authorized users.

## Background

Hepatic hemangioma (HH), hepatic metastasis (HM) and hepatocellular carcinoma (HCC) are regarded as the main reasons of liver lesions [1]. Clinically, single liver lesions without symptoms are difficult to diagnosis. Imaging examination, especially MRI, is viewed as one of the most sensitive and specific techniques for evaluating liver lesions. Diagnosis of these lesions is usually performed by visual inspection characterizing the medical imaging features such as lesion size, signal intensity and signal enhancement [2–5]. However, the hallmarks of the three types of liver lesions on MRI images overlap each other [3], especially for single lesions in the liver. The subjective nature of many diagnostic decisions related to the characterization of hepatic lesions also decreases the sensitivity of diagnosis. As a result, clinicians continuously seek better methods for accurate diagnosis.

One possible remedy for these limitations is texture analysis (TA) of MRI. TA is not a new technique and has been studied for medical imaging since 1973. More recently, TA has been applied to CT, MR and PET studies [6–8]. While the human eye cannot inspect some subtle differences in image information (coarseness, rough and busyness), the TA technique can provide a great deal of help. TA of medical images provides a quantitative measure of the imaging features that may relate to the characteristics of the pathological information in the lesions [9]. Preliminary studies of TA applications have focused on various tumors [10] where the TA had been demonstrated to improve the characterization and diagnosis. Georgiadis [11] showed that TA based on brain MRI could discriminate between metastases, gliomas and meningiomas. Holli [12] indicated that MRI texture analysis could differentiate breast cancer from normal tissue and might be able to distinguish between histological types (lobular and ductal) of invasive breast cancer. Our previous study also demonstrated that TA of T1 post contrast MRI could capture features of brain metastases from four types of lung cancers. Texture features could be a new tool for oncologists to accurately diagnose the lesions and guide therapy based on the pathological image information of tumors [13].

In most cases, normal liver tissue contains lipid and chronic liver disease often induces fatty degeneration. These situations can increase the signal of liver parenchyma on T2WI, and affect the contrast between liver parenchyma lesions and normal liver tissue. The SPAIR T2-MRI can achieve fat suppression and clear imaging presentation of the pathological changes based on the inherent frequency shift between fat and water [14]. The SPAIR T2W-MRI has been widely used in the clinical setting to increase the contrast of the image and improve the diagnosis of liver disease. We hypothesize further that the textural difference among the three types of liver lesions can be detected by TA of SPAIR T2-MRI images. The purpose of this work is to determine the use of TA on SPAIR T2 images to differentiate the three types (HH, HM, and HCC) of single liver lesions.

## Methods

### Data acquisition

**Fig. 1.**
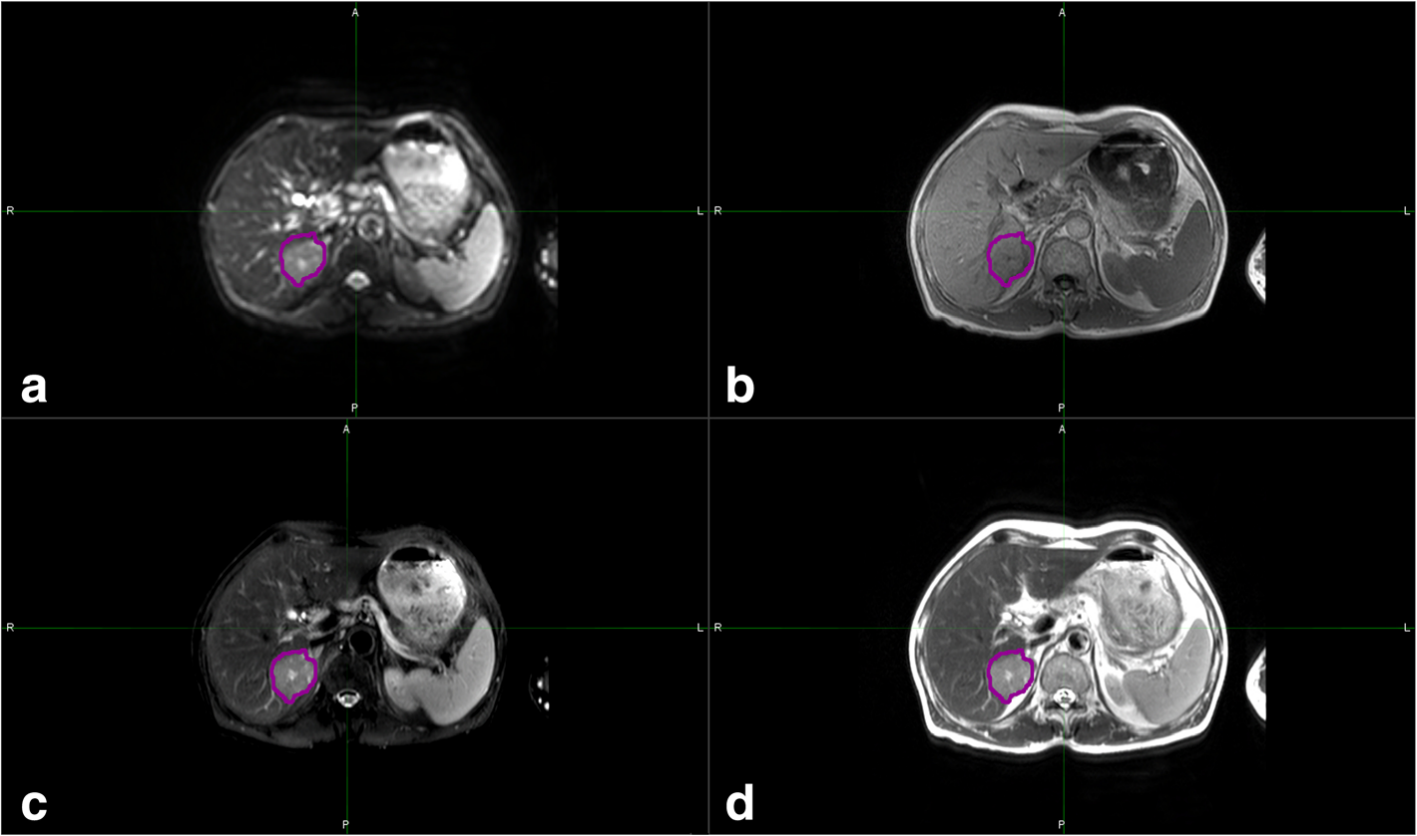
Axial slice of a patient with hepatocellular carcinoma, displayed on four different MRI series. **a** Diffusion-weighted imaging. **b** T1-weighted MRI. **c** spectral attenuated inversion-recovery T2 weighted magnetic resonance imaging. **d** T2-weighted MRI. The enhancing lesion (pink) are manually contoured using MIM software.

The SPAIR T2W-MRI datasets for 162 patients with single liver lesions were obtained from our institution from Aug 2014 to May 2016. The image datasets comprise of 55 HH, 67 HM and 40 HCC, with the diagnosis verified by postoperative pathology, biopsy or digital subtraction angiography (DSA). All scans are performed on a 3.0-T MR scanner (Philips Achieva 3.0-T X-series, Phillips Healthcare, The Netherlands) and SPAIR T2W-MRI is obtained in axial planes (slice thickness: 4 mm; slice:24; gap: 5mm; repetition time [TR]/echo time [TE], 1277.4/70 millisecond [ms]; flip angle, 233 degrees; matrix size, 256×256; field of view (FOV), 375×375mm). The four different MRI series in the same image location, including diffusion-weighted imaging, T1-weighted MRI, SPAIR T2-MRI, and T2-weighted MRI are shown in Figure 1. The figure shows that SPAIR T2-MRI can achieve more clear imaging presentation of the pathological changes than T2 weighted image. The MIM software (commercial softer ware, MIM vista Corp, Cleveland, OH) counters the same sizes of regions of interest (ROIs) for all four MRI series.

### Image pre-processing

The pre-processing step included selecting slice, contouring regions of interest (ROIs) and intensity normalization. In order to improve robustness of contouring ROIs, contours were performed by three senior board certified radiologists who specialize in abdominal imaging using a semi-automatic module on a commercially available software tool (MIM). All radiologists were blinded to the clinical information of all patients. To analyze the intra-observer reproducibility, the parameters were repeatedly measured by a second observer at an interval of 6 weeks. For each lesion, the ROI was defined and delineated around the largest cross-sectional area of the lesion inner outline. The equipment voltage changes and subtle differences in parameter setting can lead to variation of gray level intensities. In order to correct for these variations, two steps were used. The first step was filtering the image using a Wiener filter to reduce image noise and normalize tumor intensities in the same range. The second step was deciding which gray levels would be included in the range. Voxel intensity values were generally resampled in four discrete values (16-128):

1 $$ p(x)=\left[ Range\times \frac{I(x)-\underset{i\in \Theta}{ \min } i}{\underset{i\in \Theta}{ \max } i-\underset{i\in \Theta}{ \min } i+1}\right] $$

The ‘*Range*’ represents four different normalized values (16,32,64,128), *I*is the gray level intensity, and Θ is a pixels set in the ROI. Our previous studies shown that no statistically significant differences in choosing discrete values [15]. In this study, we also did some related experiments. The results showed that all textural parameters describing local heterogeneity were insensitive to the chosen discretization values, but several regional heterogeneity parameters calculated on intensity-size-zone matrices were sensitive to the chosen discretization value. The low-intensity large-zone emphasis was characterized by a mean difference of 31%±15% and 61%±18% using 16 and 128 values, respectively. On the other hand, the intensity and size variability of uniform tumor areas were largely independent (SD differences < 20%) of the discretization values, with no statistically significant differences. And several regional heterogeneity parameters calculated on intensity size–zone matrices were sensitive to the chosen discretization value, with statistically significant differences for using different discretization values (SD values > 20%). The 32 discrete values were chosen in the resampling normalization process. The detailed description for four different normalized values is shown in supporting information.

### Texture analysis

In the texture feature extraction module, many features are extracted for classification. Generally, texture contained important information which is used for the classification and analysis of many types of images. Texture features refers to the spatial relationships and arrangement of the pixels of an image. Visually, these spatial distribution and arrangement of the pixels are shown as variations in the intensity patterns or gray tone. Therefore, texture features mainly reflect gray tones of the image. The human eye can recognize texture by perceptual experience, such as roughness, periodicity, busyness and uniform, but it is quite a difficult task to feel a nice distinction for similar texture. From the SPAIR T2W-MRI images in this study, six different texture feature sets are extracted separately from intensity histogram features (IHF), gray level co-occurrence matrix (GLCM), gray level gradient co-occurrence matrix (GLGCM), gray-level run-length matrix (GLRLM), Gabor wavelet transform texture (GWTF), and intensity-size-zone matrix (ISZM) (a total of 233 features). The used texture features are briefly outlined in Table 1.

**Table 1:** TA features grouped by texture type

Texture type					
IH	GLCM	GLGCM	GLRLM(0°,45°,90°,135°)	GWTF	ISZM
Variance	Energy_mean/variance_	Small gradient emphasis	Short run emphasis	S_gabor-00	Small zone emphasis
Skewness	Entropy_mean/variance_	Large gradient emphasis	Long run emphasis	S_gabor-01	Large zone emphasis
Kurtosis	Contrast_mean/variance_	Gray inhomogeneous	Grey-Level Non-uniformity	S_gabor-02	Intensity variability
	Correlation_mean/variance_	Gradient gray inhomogeneous	Run-Length Non-uniformity	S_gabor-03	Size zone variability
	Homogeneity_mean/variance_	Gradient energy	Low Gray-Level Run emphasis	S_gabor-04	Zone percentage
	Sum Variance_mean/variance_	Mean Gray	High Gray-Level Run emphasis	...	Low intensity emphasis
	Cluster shade_mean/variance_	Mean Gradient	Short Run Low Gray-Level emphasis	S_gabor-47	High intensity emphasis
	Cluster tendency_mean/variance_	Gray variance	Short Run High Gray-Level emphasis	A_gabor-00	Low-intensity small-zone emphasis
	Inverse difference moment_mean/variance_	Gradient variance	Long Run Low Gray-Level emphasis	A_gabor-01	High-intensity small-zone emphasis
	Inverse Variance_mean/variance_	Gradient correlation	Long Run High Gray-Level emphasis	A_gabor-02	Low-intensity large-zone emphasis
		Gray entropy	Run Percentage	A_gabor-03	High-intensity large-zone emphasis
		Gradient entropy		A_gabor-04	
		Mixture entropy		A_gabor-05	
		Gradient difference moment		...	
		Gradient inverse difference moment		A_gabor-47	

5 numbers of wavelet scales, 8 numbers of filter orientations, =0,1,2,3,4 and =0,1,2,3,4,5,6,7

S_gabor-00 represents mean square energy features of *ν*=0 and *μ*=0

A_gabor-01 represents mean amplitude in scale of *ν*=0 and *μ*=1

### Detailed feature description

The histogram feature of image is the one dimensional statistics value that reflects the distribution of gray-level value. The mean, variance, percentiles, skewness, kurtosis, energy and entropy are the common features of the gray-level histogram. The environment changes of equipment may inflect gray-level value, so we only select three features (variance, skewness, and kurtosis) that do not vary along with gray-level changes. The selected features show the change trend of gray-level value. Although the histogram feature has obvious advantage in showing the distribution of value, it has limitation in reflecting spatial relationships or correlations between pixels. In order to overcome the above defect, the following features are studied.

The GLCM, describing pair-wise arrangement of pixels with the same gray-level, is used to highlight local heterogeneity information. The GLCM describes the arrangement of pixels with the same gray scale. The number of pair-pixel with the same gray-level in a predefined direction and distance is counted and summarized in the matrix.

In the first step of computing the GLCM, selection of a special ‘direction’(d) and a ‘distance’(*θ*) is needed. In this study, an 8-connexity (neighboring pixels in all directions including 0^∘^ , 45^∘^ , 90^∘^ , 135^∘^and their opposite directions) and 4 distances (i.e., 1, 2, 4, 8 pixels distance) are chosen. Then each distance and direction can get the same textural parameters, but the lesion can be depicted better in a special ones. Then, 80 textual parameters (the calculated mean and variance of the four directions) are calculated according to the Haralick [16] features in order to obtain the regional isotropy properties.

The gradient can show the spatial variation between gray-level pixels in the image. A high gradient value shows a steep variation in that point, whereas a low gradient does a smooth variation. The difference of the gray level value between neighbor pixels is described as the gray level gradient of the pixel which is derived from the gray image. The GLGCM, which describes the corresponding relation between the gray value and the gradient of each pixel in predefined direction, is acquired from the original image and the corresponding gray image. Besides, 15 textual parameters highlight local variations of pixel intensities and its gradient are extracted from GLGCM according to the Haralick features [16]. Detailed features extractable from GLGCM are included in Additional file 1: Table S1.

A set of features based on gray level run lengths matrix (GLRLM) is also employed. A gray-level run-length is defined as a measure of contiguous gray levels along a specific orientation. The length of runs mainly depends on how rough the image. GLRLM depicts distribution of gray in a specific line of image. In a coarse texture, we will expect that relatively long runs occur relatively often, whereas a fine texture should contain primarily short runs. The run length features are defined as follows [17]. For example, given the special direction, GLRLM measures how many times there are runs of two or more consecutive pixels with the same value. For each ROI, we calculated the GLRLM in each one of the four 2D directions (0^∘^ , 45^∘^ , 90^∘^ , 135^∘^). Then, for each of the four directions, the same eleven descriptors can also be calculated for the GLRLM for image. The final values of per descriptor are the four values obtained from the four orientations. A summary of the features extractable from GLRLM is included in Additional file 1: Table S2.

The Gabor transform feature, a special case of the short time Fourier transform, is used to reflect spatial relationship of image in different scale and frequency domain. The wavelet analysis can be interpreted as image decomposition in a set of independent, spatially oriented frequency channels. In this study, we choose 5 numbers of wavelet scales and 8 numbers of filter orientations. 40 mean square energy parameters and 40 mean amplitude parameters are extracted.

Finally, ISZM is used to characterize the regional information of the size and intensity of pixel zones with the same gray value (homogeneous zones). Mathematic definitions of regional heterogeneity formulas use in this study are summarized in Florent Tixier [15]. The ISZM is similar to GLRLM encoding algorithm; the difference is that GLRLM reflected gray variation in each one of the four directions (0^∘^ , 45^∘^ , 90^∘^ , 135^∘^), while the ISZM characterizes the regional information and intensity of pixel zones with the same gray value. Detailed features extractable from GLRLM are shown in Additional file 1: Table S3.

233 textual parameters which include 142 parameters representing the local gray level variations (IHF, GLCM, GLGCM and GLRLM), 80 parameters describing the spatial relationship of image (GWTF) and 11 parameters charactering regional pixel arrangements (ISZM) are extracted from the 4 different texture matrices and Gabor transformation. Table 1 shows the detailed results.

### Statistical analysis

Statistical analysis is performed using SPSS19.0 (IBM, Armonk, New York, United States) for Windows. The differences of each feature in pair-wise comparison of the three subsets (HH, HM, and HCC) are investigated using Kruskal-Walls test. A P<0.05 is considered statistically significant. We use receiver operating characteristic (ROC) curve and the area under the curve (AUC) analysis to assess the discriminatory power of significant texture features in differentiating three subtypes.

### Feature selection methods

In this study, a large number of features (233 features for six categories) are selected. Three main important reasons to do features filter include reducing the model’s training time, improving the robustness of the model and enhancing the model’s reliability and behavior. Note that not all the features need to be evaluated, as many features are low repeatability and high redundancy.

The chosen parameters had three properties, reproducibility, high degree of differentiation and low redundancy. To assess the texture features reproducibility, we obtained test-retest scans from 15 independent patients. The reproducibility of feature parameters was an important property in repeated experiments. The concordance correlation coefficient (CCC) can meet the above purpose with value >=0.9 in this study. Another consideration was selecting the features with a high degree of differentiation, using a defined “dynamic range” (DR) metric. Similar to CCC, DR>=0.9 implied that the feature had a large dynamic range [18]. The R2 values close to 1 mean the features are correlated parameters. The procedure is repeated recursively to cover all the features. We also compute the R^2^ between the remaining features to quantify the dependency. For the above reasons, it is necessary to reduce the number of features to provide a reliable feature set, which will be used for texture discrimination and classification. Using the above methods, 38 texture parameters were generated with highly reproducibility.

### Classifiers Models analysis

All classifiers are implemented using R package caret v (6.0-71) [19], allowing accessibility with many machine learning algorithms. R package caret is a set of functions that attempted to streamline the process for creating predictive models. K-nearest neighbor (KNN, the K is the neighbor numbers, the 5 neighbors were chosen in this study; distance metric was Euclidean distance) classifier model, Back propagation artificial neural network (BP-ANN) classifier model (the number of hidden layers was 1.), support vector machine (SVM, the SVM type was C-SCM; the kernel was RBF) and Logistic regression are used for improving accuracy for classifier [20].

The classification method can potentially affect the stability of the models. The relative standard deviation (RSD) and a bootstrap approach are used to quantify stability of a classifier model [20]. The stability measure method is first proposed by Yu et al. [21]. We first selected 38 representative features based the above feature selection method and used them to compute the classifier stability. For each classification method, we all did the train step (half of training cohort) and validate step (the other half of training cohort). The AUC value was used to describe the performance on the validation step. For each feature selection method, the bootstrap approach was done in the training cohort’s subsample and reported the median±std values in the results. RSD is usually used to characterize the model’s stability. The follow equation defines it

2 $$ RSD=\frac{\sigma_{AUC}}{\mu_{AUC}}\ast 100 $$

where *σ*_*AUC*_ and *μ*_*AUC*_ are the standard deviation and mean of the AUC values respectively. The higher stability models have relatively lower RSD values than lower ones [22]. Classifiers were trained using the 10 fold cross validation of training cohort (the 112 patients) and their predictive performance was evaluated in the validation cohort (the 50 patients) using area under ROC curve (AUC).

The R software (R Core Team, Vienna, Austria, version 3.2.3) and Matlab R2013b (Mathworks, Natick, Massachusetts, USA) did all the analysis.

## Results

**Fig. 2.**
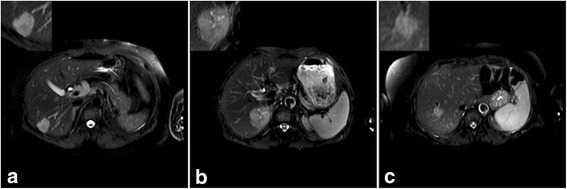
SPAIR T2W-MRIs from patients with **a** hepatic hemangioma, **b** hepatocellular carcinoma, **c** hepatic metastase. The analyzed tumor region is displayed in the top left corner

Figure 2 shows three types of MR images with HH, HM, HCC illustrating how the visual observation of different tumor can challenging.

### Classification and statistical results

As discussed above, the reproducibility of quantitative imaging texture features, CCC, DR and R2 were computed for all texture parameters. Using the above methods, 75 texture parameters were generated with highly reproducibility.

The Kruskal-Walls test was also performed to all considered features with the results showing that 9 texture parameters could differentiate between HH and HM, 16 texture parameters could differentiate between HM and HCC, and 10 parameters could differentiate between HH and HCC. The 9 texture parameters were mean of energy and homogeneity in distance 2, SRE_0/45_(the parameters of Short Run Emphasis were in 0 degree and 45 degree), SRHGE_0/45/90_ (the parameters of Short Run High Gray-Level Emphasis were in 0 degree, 45 degree and 90 degree), small zone emphasis, and high-intensity small-zone emphasis, showed a significant difference between HH and HM. To discriminate between HH and HM, we analyzed the mean of energy with ROC curves and found a cut-off of 0.89, indicating that the liver lesions whose mean of energy was higher than 0.89 were most likely from HH (sensitivity=70.6%, specificity =90.5%, AUC=0.734; P=0.014). The detailed data were shown in Table 2. Additionally, we found that there was no statistical significance between HH, HM and HCC (P>0.05). Similar results were shown in two other categories, HM and HCC, HH and HCC. A summary of detailed results is in Additional file 1: Table S4.

**Table 2:** Sensitivity and specificity for ability of textural to differentiate between HH and HM

Feature	Parameters	P	S.E.	AUC	95% CI	Sensitivity	Specificity
GLCM	Energy_mean_	0.014	0.090	0.734	56.65%-82.45%	0.706	0.905
	Homogeneity_mean_	0.012	0.075	0.782	64.23%-88.32%	0.619	0.941
GLRLM	SRE_0/45_	0.016	0.062	0.825	61.45%-84.72%	0.933	0.667
		0.021	0.057	0.812	62.12%-86.32%	0.824	0.867
	SRHGE_0/45/90_	0.012	0.068	0.801	61.45%-84.72%	0.762	0.867
		0.024	0.059	0.822	62.25%-87.33%	0.813	0.762
		0.016	0.102	0.792	58.63%-84.36%	0.922	
ISZM	Small zone emphasis	0.009	0.074	0.851	73.23%-88.23%	0.932	0.762
	High-intensity small-Zone emphais	0.007	0.067	0.832	71.23%-85.35%	0.810	0.733

Abbreviations: *AUC* area under the curve, *CI* confidence interval, *SRE* Short Run Emphasis, *SRHGE* Short Run High Gray-Level Emphasis. SRE_0/45/90/135_ and SRHGE_0/45/90/135_ depicted the parameters of SRE and SRHGE in four directions, for SRE_0/45_, two directions (0 degree and 45 degree) were calculated for SRE

After further processing of the above results, the vector dimension was further reduced. To further evaluate the models’ accuracies, the KNN, BP-ANN, SVM and Logistic regression models were used. The RSD, K-fold cross-validation and MCC evaluated classifier models stability and accuracies. Results are shown in Table 3. By inspecting the results illustrated in Table 3, we can see that the BP-ANN model displayed the highest classification accuracy over the other models in the three groups (HH and HM; HM and HCC; HH and HCC), and other classifier models had no obvious difference. The Logistic regression model had a relatively higher stability with a lower RSD value than other methods. The artificial judgment did not show better results than other models.

**Table 3:** Summary of classification results obtained by 10 fold cross-validation on three classification groups by BP-ANN, KNN, SVM and logistic regression model

Models	HH VS.HM						HM VS. HCC						HH VS. HCC					
	Acc%	Sens %	Spec %	Mcc	RSD %	AUC %	Acc %	Sens %	Spec %	Mcc	RSD %	AUC %	Acc %	Sens %	Spec %	Mcc	RSD %	AUC %
BP-ANN	88	89	86	0.86	5.5	89	90	97	92	0.79	5.8	91	90	96	85	0.80	6.4	91
KNN	85	86	85	0.75	3.8	86	87	87	88	0.76	4.6	88	85	57	90	0.77	4.3	83
SVM	83	81	86	0.75	6.1	84	83	70	93	0.77	6.0	84	88	42	95	0.83	5.2	86
Logistic regression	79	81	78	0.83	2.6	80	87	87	88	0.73	1.9	85	88	86	90	0.78	3.8	89
Radiologist	87	81	86	0.76	-	-	90	96	85	0.78	-	-	92	100	85	0.70	-	-

Abbreviations: *BP-ANN* back-propagation artificial neural network, *KNN* K-nearest neighbor, *SVM* Support vector machine, *HH* hepatic hemangioma, *HM* hepatic metastases, *HCC* hepatocellular carcinoma. Area Under the ROC Curve, Accuracy, Sensitivity, Specificity and relative standard deviation are denoted as AUC, Acc, Sens, Spec and RSD respectively

## Discussion

The image attributes usually include gray intensity, morphology and texture. Image texture feature can be defined as the spatial arrangement of pixel intensities in an image and quantitated in mathematical. However, human visual assessment of texture is usually subjective. In addition, it is difficult for the human observer to quantitate textural patterns, whereas the TA methods can provide an effective method to depict these textures [23, 24]. The feasibility of texture analysis in the evaluation of liver disease based on various imaging features have also been widely explored in computed tomography (CT), MRI and ultrasound [25, 26]. Raman [27] showed that texture analysis of CT images could distinguish the 3 liver lesion types (focal nodular hyperplasia, hepatic adenomas and hepatocellular carcinomas) from normal liver with high predicted classification performance accuracy. Mayerhoefer [26] showed that texture analysis based on MR images could classify liver cysts and hemangiomas. Xian [28] performed texture analysis based on ultrasonography images to identify malignant and benign liver tumors. This study analyzed the usefulness of texture analysis based on SPAIR T2W-MRI images in classification of the three subtypes of single liver lesions (HH, HM and HCC) and showed the potential role of textural parameters in accurate detection and diagnosis of liver lesions. To our knowledge, this is the first study to focus on the role of texture analysis based on SPAIR T2W-MRI images in classification of a small subset (HH, HM, and HCC) of the single liver lesion. As a noninvasive inspection, the textural analysis based on SPAIR T2WI-MRI images deserves further exploration. In clinical trials of liver lesions, different pathological categories need special treatment methods. If we can be accurate in classifying certain ones, the treatment of the liver lesions can be individualized and more effective.

The results demonstrate the possibility of using TA based on SPAIR T2W-MRI to differentiate different pathological types of liver lesions. The IHF feature, the GLCM feature, GLGCM feature, Gabor wavelet feature, and ISZM feature analyzed have been applied in other oncology studies as well [8, 29–31]. Global features are extracted from the intensity histogram of the tumor region, whereas GLCM, GLGCM, GLRLM, ISZM textures are matrix-based features. The GLCM, which described pair-wise arrangement of voxels with the same gray value, was used to highlight local heterogeneity information. In this study, we only find the statistical significance in distance 2, which characterize local tumor area heterogeneity. As the above mentioned texture parameters, such as Energy_mean (2)_, Homogeneity_mean (2)_ can be applied to differentiate HH and HM and Homogeneity_mean (2)_, Inverse difference moment_mean (2)_ and Inverse Variance_mean (2)_ can be applied to differentiate HM and HCC. Moreover, the Contrast_mean (2)_ and Inverse Variance_mean (2)_ can serve as quantitative indexes to classify HH and HCC. The GLGCM texture features charted the variation of area gray value. The GLGCM was acquired from the original image and the corresponding gray gradient image, and used to describe the dependency relationship between the gray value and the gradient of each voxel in predefined direction. The image gradient shows gray intensity variation. The small gradient emphasis is dominant in the homogeneous image. On the contrary, to coarse image, the value of the large gradient emphasis is dominant. Small gradient emphasis and gradient nonhomogeneity not only can classify HH and HCC but can also differentiate between HM and HCC. Meanwhile, some parameters (large gradient emphasis and gradient entropy) have high discriminatory power in between HM and HCC.

The GLRLM is a way of charactering the image, always across a predefined direction, for set of pixels having the same gray-level value. The short-run emphasis is a measure of the proportions of runs that have short lengths. It will have large values in coarse textures. The short run high gray-level emphasis measures the correlated relationship between short runs and high gray level values. The SRE (short run emphasis), SRHGE (short run high gray-level emphasis) in different directions (two directions were 0 degree and 45 degree for SRE; three directions 0 degree, 45 degree and 90 degree for SRHGE ) can differentiate HH and HM. The LRE (long run emphasis) and LRLGLE (Long Run Low Gray-Level emphasis) in different directions (two directions were 0 degree and 90 degree for LRE; three directions 0 degree, 45 degree and 135 degree for LRLGLE) can different HM and HCC. The LRE which is in two directions (0 degree and 45 degree) can different HH and HCC.

The above texture features mainly depict the dependencies of pixels in a 2D geometry space. The Gabor transform can get image features in frequency domain. The different frequencies and orientations of Gabor filters can extract meaningful texture features from image [32]. In this study, A_gabor-13, 15, 23 (A_gabor-13, amplitude in scale of *ν*=1 and *μ*=3) has the ability to differentiate HM and HCC. Some other parameters (e.g., A_gabor-22, -23) have the same capability about HH and HCC.

Additional file 1: Table S5 summarizes the features corresponding to variability in the area size or gray intensity of homogeneous areas in detail, which can also be indicators of regional tumor heterogeneity. These parameters highlight the joint distribution of intensity values and region sizes within the tumor.

These results might be attributed to the ability of texture analysis to indirectly capture the microscopic features of these lesions, which was completely different for each entity. For example, HH was composed of multiple vascular channels lined by a single layer of endothelial cells supported by a thin fibrous stroma and HCC was malignant tumor with the tumor cells demonstrated marked cytological atypia and irregular distribution [33]. These pathological features could not by visual inspection of the image of the tissue, whereas they might be presented as the variety in the arrangement of pixels which might be detected by texture analysis on medical images.

To compare diverse classifier model performance and obtain more robust classification model, four models of KNN, BP-ANN, SVM and Logistic regression were used. The k-fold cross validation, MCC and RSD ensure the reliability and stability of the model. The results showed TA can be a valuable clinical technique to distinguish various liver lesions. The same TA method was also used on three other series,but the SPAIR T2-MRI sequences obtained more robust results. The reason may be that fatty degeneration can increase the signal of liver parenchyma on T2WI and affect the contrast between liver parenchyma lesions and normal liver tissue, there is no advantage in the other three series. The only use of 2D slice has some limitations compared to 3D TA methods. Because the 2D slice might not be sufficient to capture any heterogeneities present across the tumor volume. In addition, 3D slice can capture inter-slice features that are completely ignored in the traditional 2D approach. Because the slice gap and slice thickness are too large, this study is not suitable for 3D TA methods. In our future study, the 3D TA methods will be applied.

## Conclusions

The differences in gene expression conduce the pathological types. Texture features are the outward manifestation of pathological types. The TA can quantize the arrangement of tumor cells with different pathological types. The differences of texture may be related to tumor gene expression and biological behavior. Our study gives a new method to differentiate liver pathological types using SPAIR MR images. Our study is limited by several factors, including the retrospective nature of the assessment of a relatively small group of patients. Therefore, the predictive accuracy and stability of the textural parameters should be validated in a larger, prospective patient cohort. Multi-center research in the future may also be needed. With prospective cohorts, we expect higher reliability would be gained in future studies.

## Additional file

Additional file 1: The description of the appendix. The appendix includes the supporting information for the study. It includes detailed description for features formula and other statistical results. (DOCX 1179 kb)

## Abbreviations

A_gabor-13: Amplitude in scale of =1 and =3 3D Three dimensional
ANOVA: Analysis of variance
AUC: The area under the curve
BP-ANN: Back propagation artificial neural network
CCC: Concordance correlation coefficient
CT: Computed tomography
DR: Dynamic range
DSA: Digital subtraction angiography
Energymean(2): The mean of energy in distance 2
Energyvariance(2): The variance of energy in distance 2
FOV: Field of view
GLCM: Gray level co-occurrence matrix
GLGCM: Gray level gradient co-occurrence matrix
GLRLM: Gray-level run-length matrix
GWTF: Gabor wavelet transform
HCC: Hepatocellular carcinoma
HH: Hepatic hemangioma
HM: Hepatic metastases
IHF: Intensity histogram feature
ISZM: Intensity-size-zone matrix
KNN: K-nearest neighbor
kPCA: Kernel principal component analysis
LRE: Long run emphasis
LRLGLE: Long run low gray-level emphasis
MCC: Matthews correlation coefficient
ROC: Receiver operating characteristic
ROI: Regions of interest
RSD: Relative standard deviation
SPAIR T2W-MRI: Spectral attenuated inversion-recovery T2 weighted magnetic resonance imaging
SRE: Short run emphasis
SRHGE: Short run high gray-level emphasis
std-ROI: Standard ROI
SVM: Support vector machine
TA: Texture analysis
