# Supplementary material for: Texture-based classification of different single liver lesion based on SPAIR T2W MRI images
Source: BMC Med Imaging. 2017 Jul 13;17:42. doi: 10.1186/s12880-017-0212-x (PMC5508617; doi:10.1186/s12880-017-0212-x)
Supplement: Additional file 1: — The description of the appendix. The appendix includes the supporting information for the study. It includes detailed description for features formula and other statistical results. (DOCX 1179 kb) [file 12880_2017_212_MOESM1_ESM.docx]

Table S1 A summary of textural features extractable from Gray-level gradient co-occurrence matrix (GLGCM)

| Feature | Formula |
| --- | --- |
| Small gradient Emphasis | $\left( \sum_{i=1}^{N} \sum_{j=1}^{N} \frac{H\left( i,j \right)}{j^{2}} \right)/\left( \sum_{i=1}^{N} \sum_{j=1}^{N} H(i,j) \right)$ |
| Large gradient Emphasis | $\left( \sum_{i=1}^{N} \sum_{j=1}^{N} i^{2}H(i,j) \right)/\left( \sum_{i=1}^{N} \sum_{j=1}^{N} H(i,j) \right)$ |
| Gray inhomogeneous | $\left( \sum_{i=1}^{N} \left[ \sum_{j=1}^{N} H(i,j) \right]^{2} \right)/\left( \sum_{i=1}^{N} \sum_{j=1}^{N} H(i,j) \right)$ |
| Gradient gray inhomogeneous | $\left( \sum_{j=1}^{N} \left[ \sum_{i=1}^{N} H(i,j) \right]^{2} \right)/\left( \sum_{i=1}^{N} \sum_{j=1}^{N} H(i,j) \right)$ |
| Gradient energy | $\sum_{i=1}^{N} \sum_{j=1}^{N} \left[ H(i,j) \right]^{2}$ |
| Mean Gray | $\sum_{i=1}^{N} i\left[ \sum_{j=1}^{N} H(i,j) \right]$ |
| Mean Gradient | $\sum_{j=1}^{N} j\left[ \sum_{i=1}^{N} H(i,j) \right]$ |
| Gray variance | $\left\{ \sum_{i=1}^{N} \left( i-\mu_{1} \right)^{2}\left[ \sum_{j=1}^{N} H(i,j) \right] \right\}^{1/2}$ |
| Gradient variance | $\left\{ \sum_{j=1}^{N} \left( j-\mu_{2} \right)^{2}\left[ \sum_{i=1}^{N} H(i,j) \right] \right\}^{1/2}$ |
| Gradient correlation | $\frac{1}{\sigma_{1}\sigma_{2}}\sum_{i=1}^{N} \sum_{j=1}^{N} \left( i-\mu_{1} \right)\left( j-\mu_{2} \right)H(i,j)$ |
| Gray entropy | $-\left\{ \sum_{i=1}^{N} \left[ \sum_{j=1}^{N} H(i,j) \right]\cdot log\left[ \sum_{j=1}^{N} H(i,j) \right] \right\}$ |
| Gradient entropy | $-\left\{ \sum_{j=1}^{N} \left[ \sum_{i=1}^{N} H(i,j) \right]\cdot log\left[ \sum_{i=1}^{N} H(i,j) \right] \right\}$ |
| Mixture entropy | $-\sum_{i=1}^{N} \sum_{j=1}^{N} H(i,j)\cdot logH(i,j)$ |
| Gradient difference moment | $\sum_{i=1}^{N} \sum_{j=1}^{N} \left( i-j \right)^{2}H(i,j)$ |
| Gradient inverse difference moment | $\sum_{i=1}^{N} \sum_{j=1}^{N} 1/{1+\left( i-j \right)^{2}}H(i,j)$ |

Table S2 A summary of textural features extractable from gray-level run-length matrix (GLRLM)

| Feature | Importance | Formula |
| --- | --- | --- |
| Short Run Emphasis  (SRE) | Measure of the proportions of runs that have short lengths. Expected to have large values in coarse textures. | $\frac{\left( \sum_{i=1}^{Ng} \sum_{j=1}^{Nr} \frac{p\left( i,j \right)}{j^{2}} \right)}{C}$ |
| Long Run Emphasis  (LRE) | Measure of distributions of long runs. Assumes high values for smooth textures. | $\frac{\left( \sum_{i=1}^{Ng} \sum_{j=1}^{Nr} p\left( i,j \right)j^{2} \right)}{C}$ |
| Low Gray-Level  Run Emphasis  (LGRE) | Measures the distribution of low gray level values. The LGRE is expected large for the image with low gray level values. | $\frac{\left( \sum_{i=1}^{Ng} \sum_{j=1}^{Nr} \frac{p\left( i,j \right)}{i^{2}} \right)}{C}$ |
| High Gray-Level  Run Emphasis (HGRE) | Measures the distribution of high gray level values. The HGRE is expected large for the image with high gray level values. | $\frac{\left( \sum_{i=1}^{Ng} \sum_{j=1}^{Nr} p\left( i,j \right)i^{2} \right)}{C}$ |
| Short Run Low Gray-Level Emphasis(SRLGE) | Measures the joint distribution of short runs and low gray level values. | $\frac{\left( \sum_{i=1}^{Ng} \sum_{j=1}^{Nr} \frac{p\left( i,j \right)}{i^{2}j^{2}} \right)}{C}$ |
| Short Run High Gray-Level Emphasis(SRHGE) | Measures the joint distribution of short runs and high gray level values. | $\frac{\left( \sum_{i=1}^{Ng} \sum_{j=1}^{Nr} \frac{p\left( i,j \right)i^{2}}{j^{2}} \right)}{C}$ |
| Long Run Low Gray-Level emphasis(LRLGE) | Measures the joint distribution of long runs and low gray level values. | $\frac{\left( \sum_{i=1}^{Ng} \sum_{j=1}^{Nr} \frac{p\left( i,j \right)j^{2}}{i^{2}} \right)}{C}$ |
| Long Run High Gray-Level emphasis(LRHGE) | Measures the joint distribution of long runs and high gray level values . | $\frac{\left( \sum_{i=1}^{Ng} \sum_{j=1}^{Nr} p\left( i,j \right)i^{2}j^{2} \right)}{C}$ |
| Grey-Level Non-uniformity  (GLNU) | Measure that assumes low values when runs are uniformly distributed along grey-levels. | $\frac{\left( \sum_{i=1}^{Ng} (\sum_{j=1}^{Nr} {p\left( i,j \right)}^{2} \right))}{C}$ |
| Run-Length  Non-uniformity (RLNU) | Measure of the degree of non-uniformity within run-lengths. | $\frac{\left( \sum_{j=1}^{Nr} (\sum_{i=1}^{Ng} {p\left( i,j \right)}^{2} \right))}{C}$ |
| Run Percentage  (RP) | This is the ratio of the total number of calculated runs to the total number of possible runs. | $\frac{\left( \sum_{i=1}^{Ng} \sum_{j=1}^{Nr} p\left( i,j \right) \right)}{P}$ |
| *Notation:*   - $p\left( i,j \right)$is the number of times there is a run of length *j* having a grey-level *i* - *N_g_* is the number of grey-levels - *N_r_* is the number of runs - Coefficient *C* is defined as $\sum_{i=1}^{Ng} \sum_{j=1}^{Nr} p(i,j)$ i.e. total number of runs in the image - *P* is the number of points in the image | | |

Table S3 Formulas for regional heterogeneity features of intensity-size-zone matrix (ISZM)

| Feature | Formula |
| --- | --- |
| Small zone Emphasis | $\frac{1}{∁}\left( \sum_{i=1}^{M} \sum_{j=1}^{N} \frac{Z\left( i,j \right)}{j^{2}} \right)$ |
| Large zone Emphasis | $\frac{1}{∁}\left( \sum_{i=1}^{M} \sum_{j=1}^{N} j^{2}\cdot Z(i,j) \right)$ |
| Intensity variability | $\frac{1}{∁}\left. \sum_{i=1}^{M} \left[ \sum_{j=1}^{N} \frac{Z\left( i,j \right)}{i^{2}} \right]^{2} \right.$ |
| Size zone variability | $\frac{1}{∁}\left. \sum_{j=1}^{M} \left[ \sum_{i=1}^{N} \frac{Z\left( i,j \right)}{j^{2}} \right]^{2} \right.$ |
| Zone percentage | $∁/{\sum_{i=1}^{M} \sum_{j=1}^{N} j^{2}\cdot Z(i,j)}$ |
| Low intensity emphasis | $\frac{1}{∁}\left. \sum_{i=1}^{M} \left. \sum_{j=1}^{N} \frac{Z(i,j)}{i^{2}} \right. \right.$ |
| High intensity emphasis | $\frac{1}{∁}\left. \sum_{i=1}^{M} \left. \sum_{j=1}^{N} i^{2}\cdot\right. \right.Z(i,j)$ |
| Low-intensity small-zone emphasis | $\frac{1}{∁}\left. \sum_{i=1}^{M} \left. \sum_{j=1}^{N} \frac{Z(i,j)}{i^{2}\cdot j^{2}} \right. \right.$ |
| High-intensity small-zone emphasis | $\frac{1}{∁}\left. \sum_{i=1}^{M} \left. \sum_{j=1}^{N} i^{2}\cdot j^{2} \right. \right.\cdot Z(i,j)$ |
| Low-intensity large-zone emphasis | $\frac{1}{∁}\left. \sum_{i=1}^{M} \left. \sum_{j=1}^{N} \frac{j^{2}Z(i,j)}{i^{2}} \right. \right.$ |
| High-intensity large-zone emphasis | $\frac{1}{∁}\left. \sum_{i=1∁}^{M} \left. \sum_{j=1}^{N} \frac{i^{2}Z(i,j)}{j^{2}} \right. \right.$ |
| $∁$ = number of homogeneous areas within tumor; Z = intensity size–zone matrix; M= discretization value; N = size of largest homogeneous area within tumor; z(i,j) = number of areas with intensity I and size j. | |
|  | |

Table S4a AUC (along with Corresponding 95% Confidence Intervals) of two class liver leisions, 16 features, it also shows p value with standard error (S.E) (hepatic metastases, HM and hepatocellular carcinoma, HCC).

| Comparison(HM vs. HCC) | P value | S.E | AUC | 95% confidence interval(%) |
| --- | --- | --- | --- | --- |
| Homogeneity_mean(2)_ | 0.001 | 0.074 | 0.775 | (0.63,0.92) |
| Inverse difference moment_mean(2)_ | 0.001 | 0.073 | 0.778 | (0.64,0.92) |
| Inverse Variance_mean(2)_ | 0.002 | 0.074 | 0.766 | (0.62,0.91) |
| small gradient emphasis | 0.002 | 0.067 | 0.822 | (0.69,0.95) |
| gradient nonhomogeneity | 0.039 | 0.085 | 0.664 | (0.50,0.83) |
| large gradient emphasis | 0.011 | 0.081 | 0.705 | (0.55,0.86) |
| gradient entropy | 0.039 | 0.085 | 0.655 | (0.49,0.82) |
| LRE_0/90_ | 0.019 | 0.083 | 0.690 | (0.53,0.85) |
|  | 0.013 | 0.081 | 0.712 | (0.55,0.87) |
| LRLGLE_0/45/135_ | 0.018 | 0.083 | 0.691 | (0.53,0.85) |
|  | 0.017 | 0.082 | 0.702 | (0.54,0.86) |
|  | 0.044 | 0.085 | 0.663 | (0.50,0.83) |
| A_gabor-13 | 0.009 | 0.079 | 0.729 | (0.57,0.88) |
| A_gabor-15 | 0.004 | 0.079 | 0.732 | (0.58,0.89) |
| A_gabor-23 | 0.005 | 0.079 | 0.733 | (0.58,0.89) |
| Intensity variability | 0.001 | 0.074 | 0.771 | (0.63,0.92) |

Table S4b AUC (along with Corresponding 95% Confidence Intervals) of two class liver leisions, 10 features, it also shows p value with standard error (S.E) (hepatic hemangioma, HH and hepatocellular carcinoma, HCC).

| Comparison(HM vs. HCC) | P value | S.E | AUC | 95% confidence interval |
| --- | --- | --- | --- | --- |
| Contrast_mean(2)_ | 0.044 | 0.088 | 0.674 | (0.50,0.85) |
| Inverse Variance_mean(2)_ | 0.035 | 0.081 | 0.707 | (0.59,0.91) |
| small gradient emphasis | 0.018 | 0.086 | 0.700 | (0.53,0.87) |
| gradient nonhomogeneity | 0.024 | 0.085 | 0.699 | (0.53,0.86) |
| LRE_0/45_ | 0.044 | 0.089 | 0.669 | (0.50,0.84) |
|  | 0.036 | 0.087 | 0.688 | (0.52,0.86) |
| A_gabor-22 | 0.036 | 0.088 | 0.677 | (0.50,0.85) |
| A_gabor-23 | 0.041 | 0.087 | 0.691 | (0.52,0.86) |
| Zone percentage | 0.044 | 0.085 | 0.664 | (0.50,0.83) |
| Size zone variability | 0.041 | 0.089 | 0.666 | (0.52,0.84) |

Table S5. The parameters to classify three types of liver lesions ( hepatic hemangioma ,HH; hepatic metastases, HM; hepatocellular carcinoma ,HCC. A_gabor-13 represents mean amplitude in scale of$\nu=1$and $\mu=3$)

|  | HH vs HM | | HM vs HCC | HH vs HCC |
| --- | --- | --- | --- | --- |
| IHF feature | none | | none | none |
| GLCM feature | energy_mean(2)_  homogeneity_mean(2)_ | | homogeneity_mean(2)_  Inverse difference moment_mean(2)_  Inverse Variance_mean(2)_ | Contrast_mean(2)_  Inverse Variance_mean(2)_ |
| GLGCM feature | | none | small gradient emphasis  gradient nonhomogeneity  large gradient emphasis  gradient entropy | small gradient emphasis  gradient nonhomogeneity |
| GLRLM feature | | SRE_0/45_  SRHGE_0/45/90_ | LRE_0/90_  LRLGLE_0/45/135_ | LRE_0/45_ |
| GWTF feature | | None | A_gabor-13,15,23 | A_Gabor-22,23 |
| ISZM feature | | Size zone emphasis  High intensity small zone emphasis | Intensity variability | Zone percentage  Size zone variability |

Abbreviations: GLCM, gray-level co-occurrence matrix; GLGCM, gray-level gradient co-occurrence matrix; GLRLM, grey-level run-length matrix; Gabor wavelet transform (GWTF), ISZM, intensity-size-zone matrix; SRE, Short Run Emphasis; SRHGE, Short Run High Gray-Level Emphasis; LRE, Long Run Emphasis; LRLGLE, Long Run Low Gray-Level emphasis. Energy_mean(2)_ depicted the parameters of mean of Energy in distance 2.


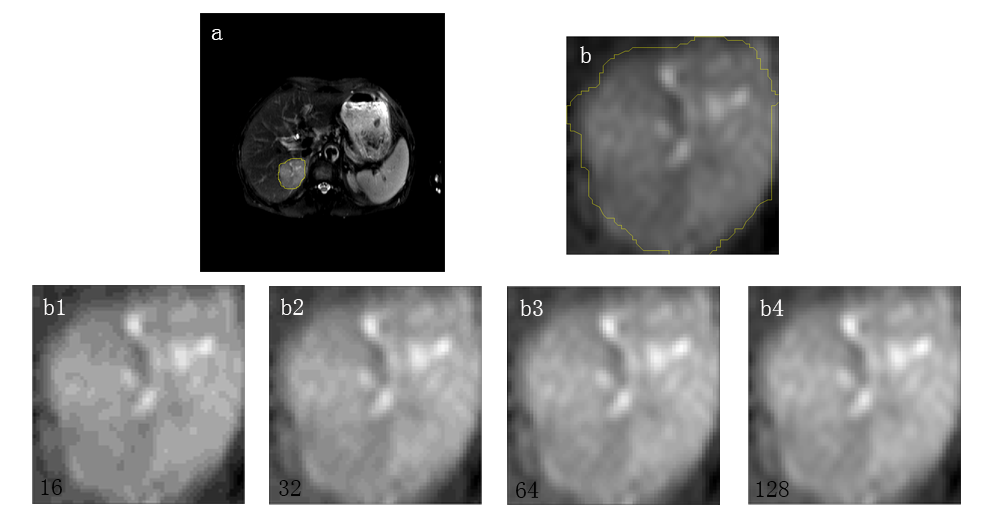


Figure 1. The figure illustrates on a tumor slice the resluting resampled ROI for each of these discretization ranges. The figure (a) shows that the private figure of axial slice for a patient with hepatocellular carcinoma; figure (b) shows the outlined area. The figure b1-b4 shows the down sampling to ranges of 16-128.


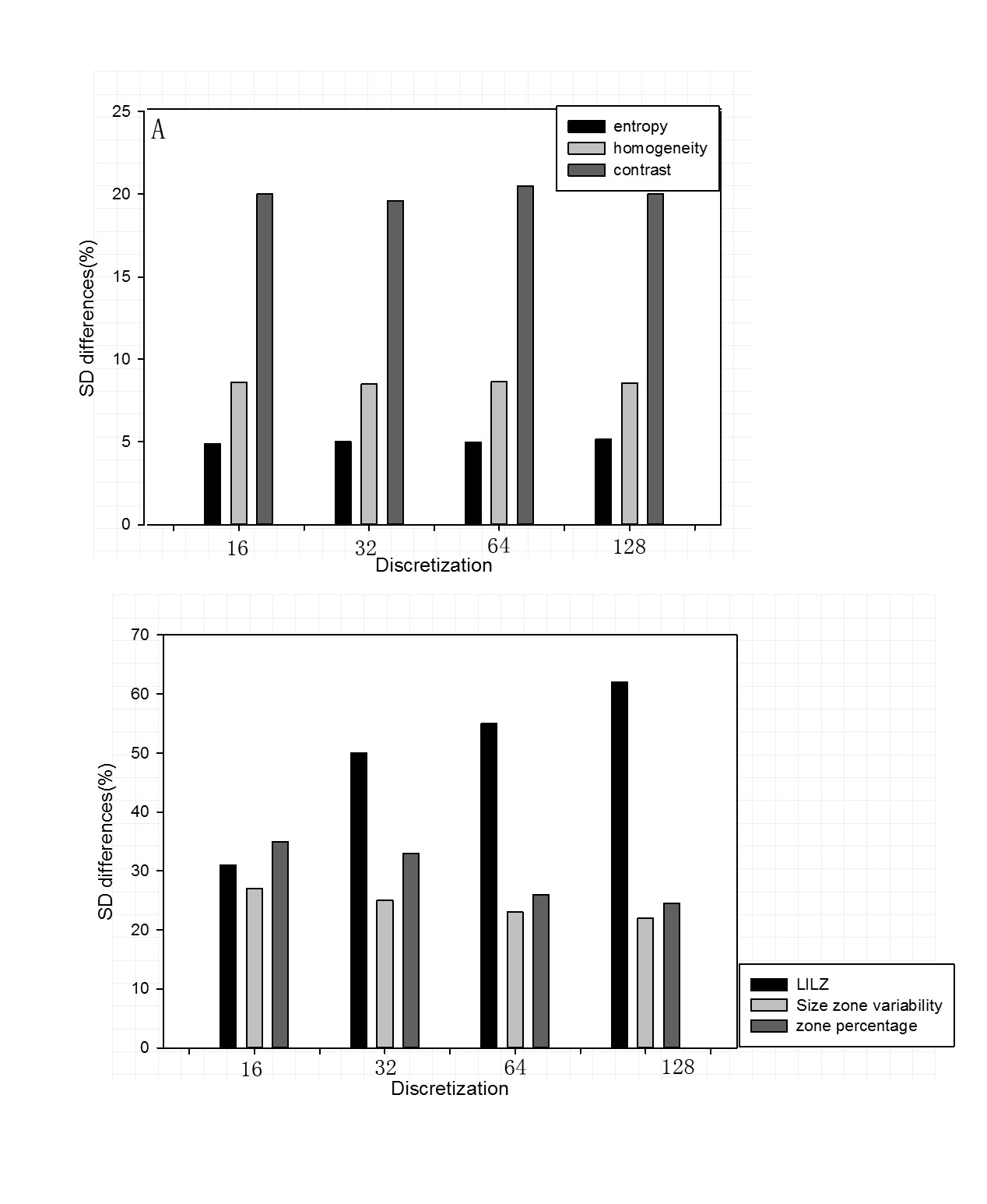


Figure 2. Plots shows the SD of mean percentage difference as characterization of discretization value for parameters derived from co-occurrence matrices(entropy, homogeneity, contrast ) (figure A showing) and intensity-size-zone matrix (figure B showing). LILZ = low-intensity large-zone emphasis.
